# Midodrine Monotherapy in Refractory Hepatorenal Syndrome-Acute Kidney Injury: A Case Report

**DOI:** 10.7759/cureus.104212

**Published:** 2026-02-25

**Authors:** Timothy J Ford, Mohamed Reffai Syed Mohamed, Rupert T Cox

**Affiliations:** 1 Department of Digestive Health, Gold Coast University Hospital, Gold Coast, AUS

**Keywords:** alcohol-related cirrhosis, hepatorenal syndrome-acute kidney injury, octreotide plus midodrine, portal hypertension, renal and hepatic failure

## Abstract

Hepatorenal syndrome-acute kidney injury (HRS-AKI) is a serious complication of advanced cirrhosis, often requiring vasoconstrictive therapy. Terlipressin with albumin is the first-line treatment; however, in cases of terlipressin failure or the unavailability of noradrenaline, alternative therapies are limited. Midodrine, typically used in combination with octreotide, has uncertain efficacy as monotherapy.

A 52-year-old woman with alcohol-related decompensated Child-Pugh B (CP-B) cirrhosis and severe portal hypertension developed HRS-AKI following a semi-elective umbilical hernia repair. Initial treatment with terlipressin and albumin led to partial resolution; however, renal function deteriorated on tapering and cessation. Noradrenaline was not feasible in the long term due to the need for central venous access, and the patient was unaccepting of long-term subcutaneous injections for octreotide + midodrine therapy. The patient was consequently started on midodrine 2.5 mg three times a day (TDS) and titrated to 10 mg TDS in conjunction with 20% albumin over the course of three days. Her renal function improved progressively over the course of two weeks, and she was discharged on midodrine 10 mg TDS. Follow-up over four months showed recovery in kidney function, and she was successfully weaned off midodrine therapy before proceeding to elective transjugular intrahepatic portosystemic shunt (TIPS) for her refractory ascites, leading to its complete resolution.

Midodrine in conjunction with octreotide is a common regimen for HRS-AKI where terlipressin or noradrenaline is not available or feasible. In our case, midodrine monotherapy improved renal function from an estimated glomerular filtration rate (eGFR) nadir of 37 to 82 in one month, remaining stable at 83 over one year later, with no recurrence of HRS post weaning of midodrine. While alpha-1 adrenergic agonists alone are not traditionally thought to overcome the splanchnic vasodilation seen in HRS, this case suggests otherwise. Our findings suggest a potential role for midodrine monotherapy as a treatment option in HRS-AKI refractory to standard vasoconstrictive therapy, where therapeutic options are limited due to the unavailability or lack of feasibility.

## Introduction

Hepatorenal syndrome-acute kidney injury (HRS-AKI) is a severe complication of cirrhosis with clinically significant portal hypertension, characterised by impairment in kidney function secondary to renal hypoperfusion [1,2]. The main pathogenic driver is splanchnic vasodilation resulting in effective arterial underfilling with the resultant compensatory activation of the renin-angiotensin-aldosterone system, vasopressin release and sympathetic nervous system activity [3]. These mechanisms initially work to preserve systemic perfusion; however, they ultimately lead to intense renal vasoconstriction, reduced renal blood flow and impaired glomerular filtration. HRS-AKI is diagnosed when a patient with cirrhosis and ascites develops AKI stage 2 or higher per the ICA-AKI criteria [4]. The European Association for the Study of the Liver (EASL) guidelines recommend the withdrawal of diuretics for 48 hours with volume expansion [2]. In the absence of shock, nephrotoxic drugs or parenchymal kidney disease and if renal function fails to improve after volume expansion and diuretic withdrawal, the diagnosis is confirmed. Terlipressin 0.85 mg is the first-line vasoconstrictor therapy for HRS-AKI, which is administered intravenously four times a day (QID) plus 20% albumin as standard of care [5,6]. Terlipressin is a synthetic vasopressin analogue preferentially targeting V1a receptors in the vascular smooth muscle, thereby increasing systemic vascular resistance [5]. The failure of terlipressin has been reported in up to 40% of patients, with acute-on-chronic liver failure (ACLF) grading being the major determinant of terlipressin responsiveness [7-11]. Noradrenaline is an alternative therapy but requires ICU-level care and central venous access [5,6]. In cases where these agents are contraindicated, midodrine, an alpha-1 adrenergic agonist with octreotide, may be used, though with reduced efficacy [5-7]. HRS-AKI refractory to medical therapy necessitates the consideration of renal replacement therapy (RRT) as a bridge to liver transplant in eligible patients [5,6]. In cases of ineligibility, patients with worsening renal function transition to end-of-life care.

Currently, there is limited evidence for midodrine monotherapy [12]. We present a case of alcohol-related cirrhosis complicated by HRS-AKI, which was successfully managed and subsequently weaned off midodrine following the lack of response to terlipressin.

## Case presentation

A 52-year-old woman with Child-Pugh B alcohol-related liver disease (CP-B ARLD) was admitted electively for large-volume paracentesis (LVP) in February 2024 for diuretic-refractory ascites. She had developed rapid fluid re-accumulation despite diuretic optimisation and recurrent AKI in the six months prior, limiting up-titration. The patient reported abstinence from alcohol, no history of hepatic encephalopathy and no varices on a recent gastroscopy performed in October 2023. She was on trimethoprim-sulfamethoxazole for spontaneous bacterial peritonitis (SBP) prophylaxis. Medications included frusemide 20 mg, spironolactone 50 mg and levetiracetam 1 g twice a day (BD). Examination revealed a large volume of ascites and a reducible umbilical hernia.

**Figure 1:**
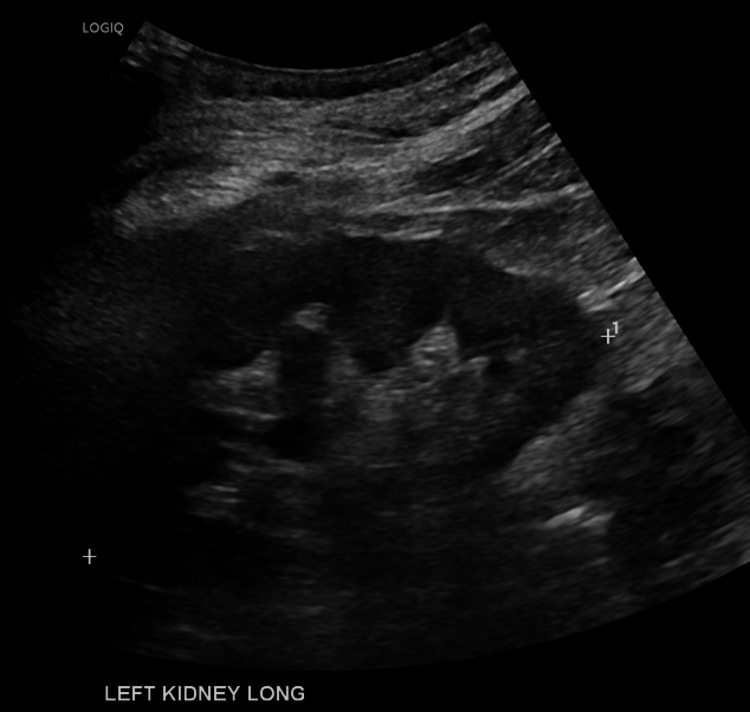
Left Renal Tract Ultrasound The left kidney showing no hydronephrosis

**Figure 2:**
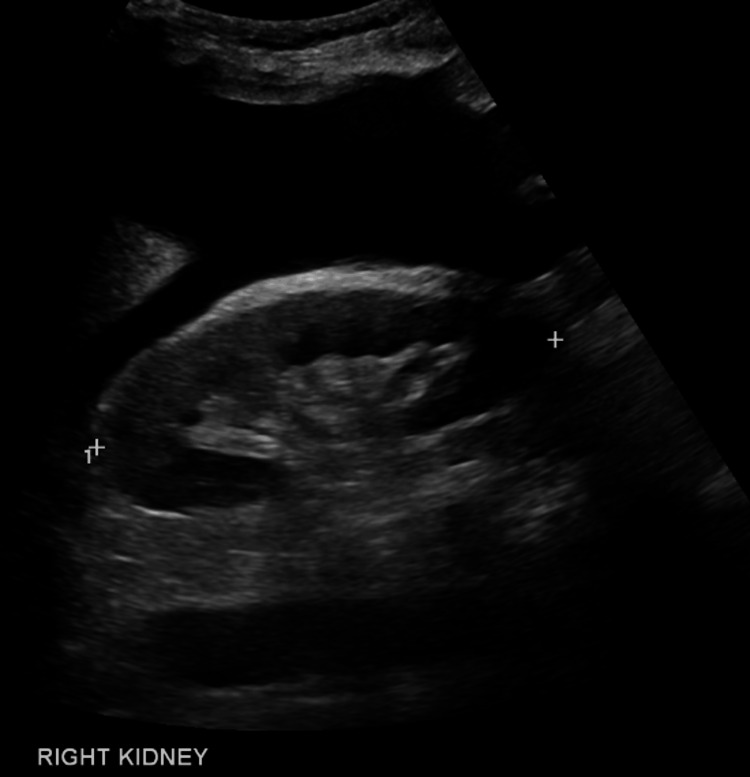
Right Renal Tract Ultrasound The right kidney showing no hydronephrosis with a moderate volume of ascites

Pre-LVP bloods showed AKI with an estimated glomerular filtration rate (eGFR) of 20 mL/minute (creatinine at 234 μmol), whereas two weeks prior, eGFR was at 72 mL/minute (creatinine at 82 μmol/L), thus fulfilling the ICA-AKI criteria. A renal ultrasound showed no obstruction (see Figures 1, 2). The patient was admitted to the hospital, diuretics were withheld and 4.5 L of ascites was drained with albumin coverage. The ascitic sample taken was negative for SBP. Her renal function improved to an eGFR of 55 mL/minute, and she was discharged three days later on 3.125 mg carvedilol. At this stage, the HRS-AKI criteria were not fulfilled. Post-renal and intrinsic renal pathology were considered unlikely given bland imaging and urinalysis.

She was readmitted five days later with a spontaneous ascitic leak through the umbilical hernia. eGFR had remained stable at 51 mL/minute with creatinine at 109 μmol/L. International normalised ratio (INR) was at 1.4 and platelets at 93 × 109/L, with liver function tests (LFTs) at baseline and alpha-fetoprotein (AFP) within normal limits. The phosphatidylethanol (PEth) level on admission was 20 μg/L (reference range: <2 μg/L); however, the PEth level one month prior was found to be 464 μg/L despite reported abstinence for 10 months. She underwent emergent umbilical hernia repair without any intraoperative or immediate surgical postoperative complications. Post surgery, the renal function declined to 31 mL/minute, and creatinine rose to 163 μmol/L despite fluid resuscitation with albumin. Urinary sodium was measured as <10 mmol/L, which is not specific, suggesting HRS-AKI. Terlipressin 0.85 mg QID was commenced.

**Figure 3:**
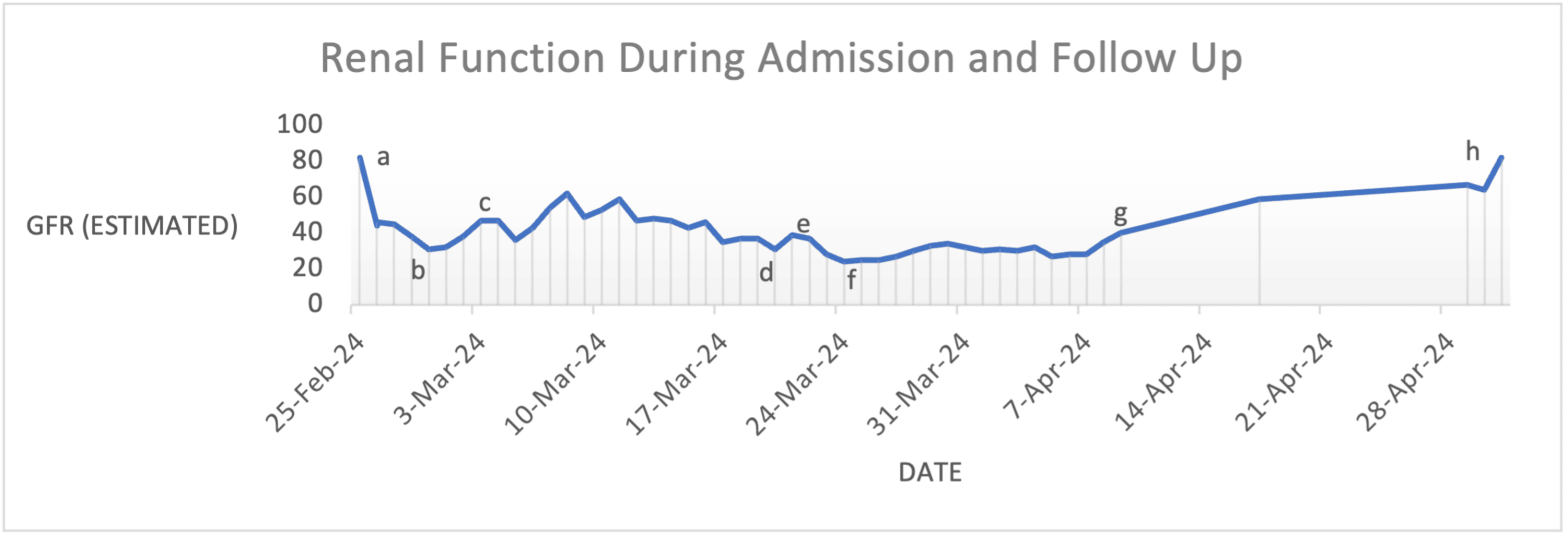
eGFR Trend During Admission and Post Discharge a, admission; b, terlipressin QID commenced; c, terlipressin weaned to TDS; d, terlipressin weaned to BD; e, terlipressin ceased and midodrine 2.5 mg TDS started; f, midodrine 10 mg TDS; g, eGFR on discharge; h, follow-up eGFR four weeks later eGFR, estimated glomerular filtration rate; QID, four times a day; TDS, three times a day; BD, twice a day

Renal function improved initially to eGFR at 47 mL/minute (creatinine at 116 μmol/L) after three days of QID terlipressin dosing. This was then reduced to three times a day (TDS), where, after one week of vasoconstrictive therapy, renal function peaked at eGFR of 59 mL/minute and creatinine of 97 μmol/L (see Figure 3). In the next week, however, renal function plateaued and then declined despite TDS therapy at an eGFR of 37 mL/minute with creatinine of 143 μmol/L. Renal replacement therapy was not deemed appropriate, given the positive PEth excluded liver transplantation. Combination therapy with octreotide and midodrine was considered; however, the patient declined subcutaneous injections despite education regarding administration and alternative strategies, and her preference was respected. Terlipressin was weaned and eventually ceased in the morning; on the same day, midodrine monotherapy was commenced at 2.5 mg TDS that evening as a last resort. Midodrine was subsequently up-titrated to 10 mg TDS. Trimethoprim-sulfamethoxazole was switched to norfloxacin to maximise renal function and avoid artificial creatinemia from the trimethoprim inhibition of the tubular secretion of creatinine. Renal function continued to improve steadily to eGFR at 40 mL/minute (creatinine at 133 μmol/L) by discharge, having been on midodrine for two-and-a-half weeks.

Outcome and follow-up

The patient’s eGFR had improved to 59 mL/minute (creatinine at 96 μmol/L) at two weeks post discharge and subsequently returned to a baseline of 82 μmol/L (creatinine at 73 μmol/L) by one month. Renal function remained stable two months later, and midodrine was weaned to 10 mg BD. Midodrine was then weaned successfully over six months. During this time, she had an episode of hepatic encephalopathy requiring ICU admission. The patient also developed AKI two weeks post weaning of midodrine from 10 mg BD to 5 mg BD without an obvious cause that improved with up-titration and albumin. Given her refractory ascites, she underwent an elective transjugular intrahepatic portosystemic shunt (TIPS) procedure, leading to the resolution of her ascites. Her current eGFR, 15 months later, is 83 mL/minute (creatinine at 72 μmol/L).

## Discussion

While vasoconstrictor therapy in combination with intravenous albumin remains the cornerstone of hepatorenal syndrome treatment, outcomes remain suboptimal, with terlipressin failure reported in up to 40% of patients [7-10]. In such cases, treatment options are limited, particularly for patients who are not suitable for intensive care or liver transplantation. Midodrine is an alternative oral alpha-1 adrenergic agonist that acts through systemic arterial vasoconstriction, resulting in a gradual increase in systemic vascular resistance and mean arterial pressure, thereby improving renal perfusion [5,6]. In contrast, terlipressin exerts rapid, potent and predominantly regional splanchnic vasoconstriction, which is associated with a higher risk of ischaemic complications, particularly with prolonged use. Consequently, midodrine is associated with a lower risk of ischaemia, albeit at the expense of weaker splanchnic vasoconstrictive effects [9]. Current guidelines therefore recommend the combined use of midodrine and octreotide to achieve synergistic haemodynamic benefit [5,6,13,14].

Our patient demonstrated a partial response to terlipressin vasoconstrictive therapy, with renal function subsequently plateauing and then declining, suggesting a partial haemodynamic benefit. The subsequent renal decline despite continued therapy raised concern for therapeutic ceiling or adverse haemodynamic effect. As a result of this, further therapeutic strategies were sought. Dialysis as a bridge to liver transplant was not considered due to a positive PEth level indicative of recent alcohol consumption, further limiting therapeutic options. Midodrine monotherapy without octreotide combined with albumin was the only available and acceptable option for the patient, with the successful reversal of kidney injury underscoring the interest in our case. Within the literature, there is a paucity of research on the use of midodrine monotherapy for the treatment of HRS-AKI refractory to terlipressin or standard vasoconstrictive therapy. Some evidence exists within retrospective studies for the reduced recurrence of AKI with midodrine monotherapy. Furthermore, a greater capacity for the reintroduction of diuretics and beta-blockers within these patients was found, highlighting potential for treatment post standard HRS-AKI therapy [15].

It is important to acknowledge the possibility of an overlap of vasoconstrictive effects. Midodrine was commenced the same day that terlipressin was ceased. In our case, terlipressin was administered in the morning, and midodrine was initiated later the same day. The reported duration of action of terlipressin is approximately six hours, with vasoconstrictive effects persisting beyond cessation, particularly following prolonged administration [16]. A residual haemodynamic effect from low-dose terlipressin overlapping with early midodrine therapy therefore cannot be excluded. However, renal function had plateaued and subsequently declined despite continued terlipressin, suggesting limited ongoing benefit. The delayed yet sustained improvement following the initiation and up-titration of midodrine supports a clinically meaningful response most consistent with midodrine’s effect. Nonetheless, a transient combined vasoconstrictive effect may have contributed to improved haemodynamics. This approach may offer complementary haemodynamic support, allowing dose-sparing and potentially fewer adverse effects than higher-dose monotherapy. The postoperative recovery and resolution of acute surgical stress are further important considerations, which may have partially influenced renal function improvement. The temporal association with the commencement of midodrine and sustained improvement, however, supports renal function recovery more strongly attributed to medical therapy.

Our case also highlights a need for a patient-centred approach in the treatment of HRS-AKI. In patients who are not deemed candidates for dialysis, liver transplant or ICU-level care, there remains a gap within care, often leading to earlier transition to supportive care. The successful use of midodrine monotherapy with albumin in our case of terlipressin-refractory HRS-AKI illuminates a need for further research and consideration of this as a therapeutic option.

## Conclusions

HRS-AKI is a serious complication of decompensated cirrhosis, which is associated with a poor prognosis and is often difficult to treat, requiring volume expansion with albumin and vasoconstrictive therapy. Currently, there is a gap in the literature with a lack of randomised trials for terlipressin-refractory cases of HRS-AKI who are not candidates for RRT as a bridge to liver transplant or not willing to undergo regular subcutaneous injections. Midodrine monotherapy with albumin may provide a viable oral vasoconstrictive alternative. Our findings, however, represent a single case study where the improvement in renal function has been multifactorial, impacted by prior vasoconstrictive exposure and progression beyond the immediate postoperative period. Broader studies are needed to evaluate the efficacy, safety and appropriate patient selection for midodrine monotherapy in HRS-AKI.
